# Performance feedback: An exploratory study to examine the acceptability and impact for interdisciplinary primary care teams

**DOI:** 10.1186/1471-2296-12-14

**Published:** 2011-03-29

**Authors:** Sharon Johnston, Michael Green, Patricia Thille, Colleen Savage, Lynn Roberts, Grant Russell, William Hogg

**Affiliations:** 1 CT Lamont Primary Health Care Research Centre, Élisabeth Bruyère Research Institute. University of Ottawa, Department of Family Medicine. Ottawa, Ontario, Canada; 2 Department of Family Medicine, and Centre for Studies in Primary Care, Queen's University. Kingston, Ontario, Canada; 3 Department of Sociology, Faculty of Arts, University of Calgary. Calgary, Alberta, Canada; 4 Centre for Health Services and Policy Research, Queen's University. Kingston, Ontario, Canada; 5 Department of Community Health and Epidemiology, Faculty of Health Sciences, Queen's University. Kingston, Ontario, Canada; 6 Southern Academic Primary Care Research Unit, School of Primary Health Care Faculty of Medicine, Nursing and Health Sciences, Monash University, Australia

## Abstract

**Background:**

This mixed methods study was designed to explore the acceptability and impact of feedback of team performance data to primary care interdisciplinary teams.

**Methods:**

Seven interdisciplinary teams were offered a one-hour, facilitated performance feedback session presenting data from a comprehensive, previously-conducted evaluation, selecting highlights such as performance on chronic disease management, access, patient satisfaction and team function.

**Results:**

Several recurrent themes emerged from participants' surveys and two rounds of interviews within three months of the feedback session. Team performance measurement and feedback was welcomed across teams and disciplines. This feedback could build the team, the culture, and the capacity for quality improvement. However, existing performance indicators do not equally reflect the role of different disciplines within an interdisciplinary team. Finally, the effect of team performance feedback on intentions to improve performance was hindered by a poor understanding of how the team could use the data.

**Conclusions:**

The findings further our understanding of how performance feedback may engage interdisciplinary team members in improving the quality of primary care and the unique challenges specific to these settings. There is a need to develop a shared sense of responsibility and agenda for quality improvement. Therefore, more efforts to develop flexible and interactive performance-reporting structures (that better reflect contributions from all team members) in which teams could specify the information and audience may assist in promoting quality improvement.

## Background

As health information systems advance, performance feedback to individual providers is becoming systematically integrated into health systems to improve care. However, improving the quality of health care is a complex challenge [1]. Research on the effectiveness of performance feedback to improve quality is mixed, and shows small to modest progress at best [2]. In the dynamic environment of primary health care reform enveloping many nations, there is still much to be learned about how new information systems and quality improvement interventions can impact patient care.

Much of the earlier primary health care research done in the 1990s on performance indicators, audit, and feedback to improve performance was done involving primarily physicians [3,4]. However, primary health care is increasingly organised and delivered through interdisciplinary teams. Given many countries have recently developed, and now regularly use, comprehensive performance indicators for primary care, researchers, providers, and policy-makers need to better understand how the emerging performance management systems, including audit and feedback, can foster quality improvement in the rapidly-changing models of interdisciplinary primary care teams. Using the theory of planned behaviour [5] to understand the impact of performance feedback to interdisciplinary teams, several factors key to newly-forming interdisciplinary primary care teams were identified that may moderate the impact of such performance feedback. A team's culture or attitude towards the performance measurement and feedback process and towards changing their practice, its understanding of the pressures to change its practice, and team members' perceived ability to control or change performance should influence the intention to change or improve current practice [6].

This mixed methods study was designed to explore the acceptability and impact of feedback of team performance data to primary care interdisciplinary teams. We sought to better understand the process of delivering performance feedback to teams, as well as the impact on intentions to improve performance of providing feedback to a whole team. Our goal was to understand if such an approach should be supported to become part of an ongoing, robust quality improvement process.

## Methods

### Participants

Seven Family Health Teams (FHTs), a primary care interdisciplinary practice model introduced in Ontario, Canada, in 2005, were recruited for an earlier study a year before this one, to validate a set of performance indicators and data collection strategy for primary care [7]. These seven FHTs varied in size, team composition, and length of time existing as a team practice. (See Table 1).

**Table 1:** Traits of participating FHTs

Type of Team	
Academic	Community-Based

5 teams	2 teams

**Range of Years in Operation** Shortest to Longest.	

3 years	20 years

**Range of Team Size** Smallest to Largest	

19 staff	67 staff

### Intervention

As part of the earlier study, in each FHT practice data was collected on a comprehensive set of performance indicators ranging from management of acute conditions to chronic disease care, practice organization and work patterns, as well as team function, using surveys administered to patients, providers, and practice managers, and patient chart audits. Data collection took place over a one week to one month period. This information was later linked to secondary administrative data. Six months to one year after that data collection had occurred, these same seven Family Health Teams were offered a single one-hour, on-site facilitated performance feedback session. These sessions presented, from the comprehensive, previously-conducted evaluation, selected highlights such as performance on chronic disease management and access indicators and patient satisfaction and team function. (See Table 2).

**Table 2:** Examples of performance indicators presented to teams

Performance Domains	Indicators presented
Chronic disease management	% patients with hypertension with chart documented blood pressure < 140/90
	
	% patients with hypertension given self-management advice
	
	% patients with CAD prescribed ASA
	
	% patients currently smoking provided information about quitting

Access	3^rd ^next available appointment
	
	Patient perception of access to after hours care for urgent issues

Patient Satisfaction	Patient satisfaction with index visit discussion with provider

Team function	Overall Team Climate Inventory score

The performance indicators presented were selected to offer feedback on outcomes and process of greatest interest to the team or greatest relevance to interdisciplinary care. The research team suggested the entire FHT team be invited to the feedback session, but allowed each site to inform and select which team members to include. A trained nurse facilitator presented each team's performance data with comparison to the mean of the seven participating FHTs for each indicator.

For most of the indicators, all the FHTs performed well or very well, with only one FHT consistently showing a performance superior to the mean across most indicators. In addition, after the session, the FHT leadership was provided with a comprehensive customized report that provided the complete results from the earlier study.

### Data collection

Evaluation of the feedback process and impact was conducted using a mixed-methods approach. Before each feedback session, participants were asked to complete the first page of a short survey and finish the rest after the session. The questions asked about individual preferences for performance feedback on content and process aspects. Data was entered into an SPSS data file and the pre- and post-mean, as well as the range of results across the seven study FHTs for each question, were calculated and also further broken down by profession. A research associate's observation notes and presenter narratives (written immediately following feedback sessions) recorded the questions of participants, dynamics of the group, and impressions about the session.

Data collection also involved two rounds of semi-structured telephone interviews with individuals from the seven participating FHTs March-June 2009. At the end of each team feedback session, volunteers were solicited for an individual telephone interview. An email invitation was subsequently sent to the team when initial response rates were low. The first round of interviews, completed in the four weeks after the feedback session, used maximum variation sampling [8] by FHT, profession, and years working at the FHT to include as diverse a sample as possible while ensuring each profession had several participants. First round interviews explored the participants' opinions about the indicators used, their experience of the feedback session, attitudes towards changing or improving their performance, and explored existing performance management systems present in the FHTs and perception of their ability to change their performance or the teams performance. Participants were provided a copy of the PowerPoint performance feedback presentation shown to their FHT in advance of the interview.

A second round of interviews, designed to allow for member checking, assessment of early impact and follow up of emerging themes, was conducted ten-to-fourteen weeks after the session. Participants had volunteered during the team presentation or identified themselves through email as agreeing to an interview. We selected critical case participants [8] (recognized change agents and allied health professionals, or perspectives omitted in the first round) to confirm or disconfirm findings. Participants in the second round interviews were provided a short summary of findings to date and the complete indicators list to review in preparation for the interview.

Question sequencing was flexible to allow participants' responses to guide the discussion. The guide was modified progressively in keeping with iterative processes of data collection and analysis, allowing insights from early interviews to inform topics discussed in subsequent interviews. Interviews were audio-taped and summarized (with key quotations transcribed verbatim).

### Data organization and analysis

Data organisation and analysis adopted an initial immersion-crystallization approach [9]. Observation notes, presenter narratives and interview summaries were reviewed in their complete form by the analytic team, which was comprised of the two principal investigators, two research associates, and the project coordinator. Key themes were identified from this data. A coding strategy was developed by the analytic team using these emerging themes, and shaped by the research questions, existing literature, and preliminary themes identified during the review of the literature. A standard qualitative approach of template-organizing style of interpretation was used to organise the data using NVIVO 8.0 software. This was followed by a second immersion/crystallization process with retrieved segments organised by nodes [10]. Weekly meetings were held to discuss emergent themes, patterns, and connections within and across summaries and node reports, and the coding strategy and data organisation were refined as needed. Each node report was reviewed by two members of the research team independently, and findings and interpretations were summarized and shared with the analytic team. The analysis process served to identify potential individual biases and to refute or clarify interpretations through consensus and ongoing reference to the data.

Four members of the research team were also members of different FHTs included in this study. However no research team member attended a performance feedback session in their own FHT, data collection was carried out by team members not affiliated with the FHTs, and the data analysis team was blinded to the FHT identities when analyzing the data. The study was approved by the Ottawa Hospital Research Ethics Board, Queens University Research Ethics Board, and the SCOHS Research Ethics Board.

## Results

The seven FHTs varied significantly in their existing performance management systems, including performance reviews and feedback, communication mechanisms to raise quality concerns, team members with official roles and dedicated time to support quality improvement activities, as well as team organization that might impact quality improvement and responses to performance feedback. Nonetheless, all seven FHTs extended the invitation to attend the feedback session to all team members including allied health professionals and clerical and management staff. The feedback sessions were well attended in each FHT with a diverse mix of disciplines represented in each FHT. See Table 3 for information about session attendance by site.

**Table 3:** Session attendance, by site

Site No.	No. staff invited to session	No. staff present on feedback day (not away/on vacation)	No. staff attended	% staff attending (# attended / #present)	No. of surveys received	Response Rate of attendees
1	67	UNK*	38	At least 57%*	33	87%

2	60	UNK*	11	At least 18%*	11	100%

3	24	22	20	91%	18	90%

4	24	17	17	100%	13	76%

5	47	35	27	77%	17	63%

6	19	15	15	100%	14	93%

7	UNK	UNK*	31	At Least 47% *	28	90%

Total			159		134	84%

^a ^: Unable to obtain the number of staff unavailable/away the day of the feedback session.

UNK: Unknown

Table 3 provides an overview of the attendance for each site

Twenty-four feedback session attendees participated in the first round of interviews. Ten FHT members participated in the second round of interviews. Table 4 shows the professional groups of interviewees.

**Table 4:** Interview participants by professional groups, round one and round two

	Round 1	Round 2
**Physicians**	6	3

**Nursing Staff (RN + RPN)**	2	2

**Nurse Practitioners**	4	1

**Pharmacists**	3	1*

**Social Workers**	2	0

**Dieticians**	3	1

**Management**	2	2*

**Administrative Support Staff**	2	0

**TOTAL**	24	10

*indicates that one individual was interviewed in both rounds of the study.

This table tallies the number of participants according to professional groups for each round.

Several recurrent themes emerged from the data analysis related to the acceptability of the performance measurement and feedback intervention as well as its impact on attitudes, subjective norms, and perceived ability to change performance. These are summarized below.

### Performance measurement and feedback to the whole team was welcomed across teams and disciplines

The use of performance data to support quality improvement processes in the FHTs was widely accepted by interview participants. Members of all disciplines across all the FHTs welcomed feedback on the whole team's performance. "*Kinda like a FHT scorecard? Yeah I think that is not a bad idea*," said one participant (nurse practitioner 1). In general, interview participants accepted the importance of performance measurement in primary care, specifically for clarifying the impact of new programs and giving direction for future initiatives. One office manager noted that for their special chronic disease management programs, *"We can use that [performance feedback received] now rather than have to say before we start any program, 'We need to benchmark where we are' " *(office manager 1).

A pharmacist added, *"If you don't have the numbers and you don't know where you are, you don't know where you need to go, you don't know where you need to devote your resources" *(pharmacist 4). One physician described the performance feedback as motivating.

*"One of the things that motivated physicians long before they ever had status or any financial, you know, earning potential, it was the ability to see yourself as performing well...physicians...like to see that they are in the top half of the class. And I think that, really, just knowing that there is a top half of the class allows people to shoot for it, no matter what you make that class to be" *(physician 7).

These interview comments were consistent with the broader survey where most participants felt team performance measurement and feedback should be done on a regular and ongoing basis either every six months or yearly. (See Table 5).

**Table 5:** How often should the team's performance be evaluated in months?

Time for Evaluations	Frequency	Percent
Monthly	2	1.7

Every 3 Months	12	9.9

Every 6 Months	40	33.1

Yearly	46	38.0

Every 2 Years	21	17.4

Survey results for all 121 respondents

This table provides frequency and percentages for the response to how often team's performance should be evaluated in months.

Participants of six of the seven FHTs expressed appreciation for the presentation of FHT level performance data at a full team meeting. This was echoed in the survey results, wherein the preferred mode of feedback to the team was use of team meetings and custom reports highlighting FHT level results. (See Table 6).

**Table 6:** How useful would the following methods of offering performance feedback be in enabling you to improve your individual performance?

Indicator (Not at all Useful = 1 to Extremely Useful = 5)	MDs (n = 32)	Nurses & NP (n = 43)	Allied Health Professionals (n = 17)	Administrative (n = 25)
Descriptive statistics of patient care processes % of patients with a BP recorded in the chart in the past year, # of patients seen, etc.	3.8 ± 0.8	3.9 ± 0.8	2.4 ± 1.1	2.8 ± 1.7

Descriptive statistics of patient care outcomes % of patients with hypertension who are well controlled, % of diabetics who are well controlled, etc.	4.1 ± 0.8	3.9 ± 0.8	3.2 ± 1.0	2.8 ± 1.8

Comparison of your FHT to others on patient care processes % of patients with a BP recorded in the chart in the past year, # of patients seen, etc.	3.5 ± 1.0	3.7 ± 1.0	2.7 ± 1.2	2.5 ± 1.7

Comparison of your FHT to others on of patient care outcomes % of patients with hypertension who are well controlled, % of diabetics who are well controlled, etc.	3.6 ± 0.9	3.7 ± 1.0	2.7 ± 1.1	2.6 ± 1.7

MDs, nurses and NPs, allied health professionals, and administrative staff rated the usefulness of each method for offering performance feedback

The exception to the general appreciation for the feedback to the group was in one FHT where interview participants suggested utilization of virtual modes of feedback (e.g., emailing the feedback). *"[I]t can be threatening to someone who has done stuff the same way for 25 years, to be told that people can measure this now and they can tell you whether you are effective or not, and their records are completely accessible for analysis" *(physician 3). In contrast, various participants noted that relying on non-interactive methods of feedback would reduce the likelihood of people engaging with the information together.

While a group presentation of team level data was acceptable, the idea of future performance data being accessible to the broader public was more controversial. Survey respondents from all disciplines ranked this as the least preferred method of receiving feedback. (See Table 6). Some interview participants however, recognized a principle of accountability to the public and funders. For instance one participant noted, *"We take a good chunk of the public's money, and it is nice for the public to know what they are getting for their dollar" *(pharmacist 1). Most interviewees stated that the provision of anonymized data to the general public was acceptable. In fact, one pharmacist felt such a transparent system would be motivating in that fear of scoring the worst in the region on an indicator would spur action: *"People are going to sit down and go, 'I don't want to be the bottom of the list and end up in the Globe and Mail on this'...What can we do about this?" *(pharmacist 5). He added, however, that if such an accountability system focused on penalization, the resultant anxiety about performance could be a barrier to productivity, morale, and recruitment to FHTs. Some participants raised concerns with publicly available performance data, saying that specific FHTs could be identified in such a system or that if taken too far, poorly presented, misinterpreted or if done with the wrong purpose, public performance data could be harmful.

A few participants cautioned against over-reliance on measurement alone. In the words of one family physician, *"...not everything that's important can be easily measured. And not everything that's easily measured is important" *(physician 3). Another physician (7) noted that guidelines are not rules: systems that measure performance through looking at attainment of targets alone can be misleading and disheartening since, in reality, many of the patients may have achieved a high level of risk reduction, but do not meet the target.

### Performance indicators did not equally reflect the role of different disciplines within an interdisciplinary team

The health care providers interviewed found the vast majority of the indicators acceptable and important to primary care. The few exceptions to this were indicators where a newer guideline had emerged since indicator selection (e.g., aspirin use for those with coronary artery disease), or those where controversy exists in the health care community (e.g., bone mineral density screening). Access and patient satisfaction indicators were those that the broadest range of FHT members saw as both reflecting their contribution to the FHT and important to the team. When specifically asked about the acceptability of the indicators presented, the relevance of the information to their work, and what they perceived was most important for future performance measurement, no interviewees raised questions about the clinical significance of the results or reported differences between FHTs.

While the health care providers interviewed agreed the indicators selected were acceptable and important for primary care, they varied as to the extent they thought the indicators presented captured their contribution to the team or were relevant to their own performance. Very few participants felt their performance could not be measured. However, on initial questioning about whether the indicators captured or reflected their work, most allied health participants commented that the indicators were overly biomedical or focused on the work of physicians, excluding non-biomedical contributions. One nurse practitioner said, *"It reflected the physicians' work quite a bit. I didn't think it reflected the NPs work as much and the other allied health professionals were almost left out" *(nurse practitioner 1).

*Similarly, one pharmacist stated, "I don't know that I could really necessarily see myself and my contributions in there" *(pharmacist 5). In addition, one registered dietician commented that the FHT-wide evaluation was not likely to show *"true meaning of everything that is going on," *and that the indicators might have missed the full range of roles in the clinic: *"I don't think you looked at the full team, I think you looked at indicators of primary care that family physicians and nurse practitioners would consider" *(registered dietician 7).

When probed further, however, some profession-specific patterns emerged. The two social workers who participated in this study saw the indicators as mostly irrelevant to their work and doubted that their individual performance and contribution to the team could be measured due to the unique problems that their patients present, and the lack of simple measures, such as those used for biomedical care. One social worker commented on the challenges of measuring the performance of certain health care professionals: *"If you were to say we were going to compare social worker to social worker, you would have to give social worker patients who were controlled in the degree of their depression or degree of their anxiety, which would be impossible to do" *(social worker 1). The lack of performance indicators for mental health presented at the group feedback sessions was noted particularly by social workers and offered as a reason their work was not reflected in the team presentation.

With more focused questions, allied health and nursing professionals noted that the indicators were partially reflective of their actual or potential contribution to patient care. Those with a nursing background saw their role reflected in prevention and chronic disease process indicators: *"The nursing role is more basic: you take the patient back to the room, vitals, brief description...a little bit of health promotion and health prevention," *(registered nurse 4). The nurse practitioners echoed this trend, with two of the four of them adding that their work is reflected in chronic disease health outcome measures. These interviewees spoke generally about process indicators, however, without identifying specific indicators that would reflect their work with more accuracy. The dieticians and pharmacists saw their work reflected more in chronic disease care process and health outcome indicators. In contrast, the physicians saw the indicators as capturing the type of work they do: "*I thought it was all applicable*" (physician 3). Some of those not participating in the previous study, which collected the performance data, noted that the sampling strategy and collection process (limited to a sample of seven physicians/site) meant that their actual performance was unmeasured.

One physician noted that he would like to better understand the role of other team members and wanted indicators that reflected this. He also would have wanted the patient perspective about "*what they got from their visit with the physician or whoever*" as patients receive different information from each provider. He felt it would "*be very interesting to have someone say it was when I saw the dietician that the penny dropped, or the doctor just checks my blood pressure and helps me out the door, but the nurse took time to listen to me*" (physician 2). Several other interviewees also noted they would have found more qualitative data capturing the patients' experiences and patients' perceptions of health valuable. This would put "*flesh on the bone of what we mean by health*" (social worker 7) in our indicators of performance. Further, survey respondents generally ranked indicators of patient satisfaction among the most helpful to lead to individual performance improvement (See Table 7).

**Table 7:** How important are the following indicators in helping you to improve your individual overall job performance?

Indicator (Not Important = 1 to Very Important = 5)	MDs	Nurses & NP	Allied Health Professionals	Administrative
Number of patients seen	3.7 ± 0.9 (n = 31)	3.3 ± 1.3 (n = 39)	3.0 ± 1.4 (n = 17)	3.1 ± 1.6 (n = 21)

Patients' satisfaction with interactions with you	4.6 ± 0.6 (n = 30)	4.2 ± 0.9 (n = 40)	4.6 ± 0.8 (n = 15)	3.8 ± 1.4 (n = 24)

Team's satisfaction with their interactions with you	4.5 ± 0.8 (n = 28)	4.3 ± 0.8 (n = 41)	4.5 ± 0.9 (n = 17)	4.0 ± 1.1 (n = 25)

Number of patients referred from other providers	2.4 ± 1.2 (n = 25)	3.2 ± 1.2 (n = 36)	3.6 ± 1.4 (n = 16)	2.1 ± 1.1 (n = 16)

Preventive health manoeuvres performed or omitted	4.7 ± 0.6 (n = 28)	4.2 ± 1.0 (n = 37)	3.1 ± 1.6 (n = 14)	3.4 ± 1.6 (n = 22)

The time to the next available appointment for a patient to see you	4.0 ± 1.0 (n = 29)	3.6 ± 1.3 (n = 39)	2.9 ± 1.3 (n = 17)	2.8 ± 1.8 (n = 20)

MDs, nurses and NPs, allied health professionals, and administrative staff rated the importance of each method for offering performance feedback

### The process of giving performance feedback to the team could build the culture of performance management, and strengthen team function

Despite the fact that all FHTs had several team members participating in a provincial quality improvement learning collaborative called the Quality Improvement and Innovation Partnership (QiiP) that required team meetings and performance measurement, participants expressed that the process of giving feedback sessions to the whole team was 'a good start' to introducing performance management concepts to everyone and building it into the culture. As one interviewee noted, "*I think every professional oughta have the desire to want to review how they're doing and improve upon it, but if that's not already engrained in the culture then, you know, maybe some help would be useful*" (pharmacist 5).

Several interviewees indicated that the feedback made them think about improvement or reinforced that they were doing a good job. For instance, one participant commented, "*It makes me think about things; you know when you are so busy doing, you aren't necessarily thinking" *(clerk 3).

Common sentiments included that the whole group session contributed to a process of building toward being, or functioning as, a team. Team feedback could cause some temporary disruption to team function, but one participant maintained that "*maybe the team needs disrupting if it gets bad feedback*" (physician 1). In addition, another interviewee stated, "*I think we're a team, and we have to own as a team our successes and failures*" (pharmacist 1). Indeed, interviewees indicated that feedback could frame a common goal as well as improve team function if results were presented: One participant recommended, "*Have the team decide what to do with the results" *(social worker 1).

### The process of giving feedback to teams increased perceived capacity to change practice

Several participants noted that bringing the whole team together for feedback would increase the whole team's capacity for quality improvement, as previous attempts to improve the quality of care informally or with just a few individuals had achieved little success. One participant in particular shared, "...*It's really hard to get everybody at the same place at the same time on the same page thinking the same thing. In other words system-wide changes require that the entire team is involved from the clerk to the doctor in examining change, and testing the change*" (pharmacist 5).

### Performance feedback should feed into the diverse existing quality improvement organization systems in each team

Despite the general sentiments that the group session strengthened the team and assisted in shaping common attitudes and beliefs about performance, many saw a need for the information to be presented differently in the future if the goal was to change practice. Preferences were expressed for individual-level data as well as data relating to the work of smaller groups either within the team or "mini teams" with a common focus.

A common sentiment articulated was that these practices were not yet a team. *"...[W]e will become a team in time" *(social worker 1). In the meantime they were not sufficiently able to come together as a group and say *" Ok what did we think about [that performance feedback] and I don't think we are there, to reflect on that and say how are we functioning as a team, we aren't enough of a team to reflect on that" *(social worker 1). However, practices were also not conceptualized as individuals working in isolation; many mentioned organizational designs that involved 'mini-teams': "*We are many teams within a team*" but not aware of what everyone is doing (physician 2). A common recommendation was to break down and present specific information to target audiences, such as the mini-teams, with a more narrow focus. These mini-teams could have a disease focus such as diabetes, or a role or intervention focus such as nursing or well-baby clinic. Another possibility would be for them to be a small subset of the FHT clinicians who work together in a consistent and integrated fashion providing more traditional primary care services.

When asked if feedback would be better delivered to professional groups within a clinic, however, one participant responded: "*Well the main challenge is each clinic works differently, and I find for us, we work a lot in a team. Even if you break it down, like, if you take all the nurses together, or all the, its not, you lose that sense of collaboration, like how we work together" *(registered dietician 5). Another responded, "*Technically, we work as a team...I don't think it is necessary*" (nurse practitioner 2). Furthermore, two physicians from the same FHT expressed an interest in team feedback being given to the physician group only. Thus, there was uncertainty and variability across FHTs as to who would be best to receive performance feedback for the team.

While it was recognized that performance measurement could improve the team as a whole, several participants gave examples of how they individually hoped to or were in the process of seeking out performance information on their own patients. Most still wanted individual feedback that reflected only their own work: "*It would have been nice to have more specific info for each provider*" (nurse practitioner 1). Individual feedback would inspire more change until the *"team feels like a team" *investing and contributing no responsibility would be taken from team feedback (pharmacist 1). This feedback was seen as part of professional development, "*because it is through feedback that we grow, we learn*" (registered nurse 4). Several participants shared that individuals can take initiative and make things happen to improve quality of care more effectively than waiting for a common FHT vision or action plan or as one interviewee stated, *"That is the way it is done, by individual interest" *(pharmacist 1).

### The effect of team performance feedback on intentions to improve performance was hindered by a poor understanding of how team could use the data

Three months after the feedback sessions, those interviewed who were outside of the management structures within the organizations expected the feedback data to be utilized in future meetings or used by specific committees within the FHT to guide priorities or improve practice. Regardless of this expectation, none of the participants could actually identify how the feedback had been, or would be, used in planning or practice change. In several FHTs, leaders interviewed attributed the limited use or review of the performance data to their being early in their performance management skill development, or to its confirming nature of already-known problems. In two sites, however, the feedback conflicted with existing priorities or perceptions of weaknesses such as poor access; in both cases, the feedback results were dismissed and the team's priorities did not change.

## Discussion

Many elements are involved in improving the performance of primary care teams at the individual, team, organisation, and health system and surrounding community levels [11-15]. Many different interventions to change providers' behaviours have targeted one or several of these components and have shown that multifaceted approaches are more likely to improve performance [11,16]. Audit and feedback of team performance as an intervention for interdisciplinary teams may have a unique role in fostering quality improvement as part of a multifaceted approach. Team feedback was welcomed by participants from all the disciplines in this study and was seen as useful, necessary, and potentially motivational. This intervention was also seen as having the potential to shape team culture or attitudes as well as to enable the establishment of common goals and understanding of performance standards. These are valuable precursors to building the intention to change practice [5] and improve performance among teams, one of the first steps in quality improvement efforts.

Additionally, team performance feedback was perceived as having a potentially enabling effect on the team's ability to change practice by bringing people together to focus on performance. The opportunity to process performance results together has been shown to be valuable to groups of physicians [17] enabling quality improvement [18]. The opportunity to come together for a similar purpose may be a valuable tool for primary care interdisciplinary teams as well. This intervention, however, did not allow significant time to process results as a team or actually establish common goals as most of the meeting was devoted to the presentation of results. Follow up interviews identified that the lack of clarity about who would or should use the information presented to initiate practice change is a significant barrier to practice improvement. More time for the team to review the results presented might have enabled actual goal setting and identification of responsible team members to initiate change.

Previous research suggests that performance feedback should integrate into existing performance improvement systems for best effect on improving quality [2,19]. Several participants from different teams suggested that smaller teams, with a narrow focus such as diabetes care or nursing care, would be a more functional unit for receiving and acting on performance data. However, these existing systems seemed to differ in each team. Efforts to improve the quality of care in multidisciplinary teams might need to be sensitive to the existing diverse organizational structures and leadership cultures. Perhaps efforts to assist teams in an a priori manner to identify or build the functional groups to receive and act on performance data on their priorities of interest might enable better targeting and feedback to the groups with the ability to change practice [20].

Several participants expressed a preference for individual level feedback. This preference coupled with the notion that individuals can still initiate change better than or faster than the team, as expressed by several participants, may reflect the fact that team performance feedback, particularly a single session with insufficient time to process, did not sufficiently change the perception of the team's ability and intention to act on the data. While team feedback is a "good start" it is likely not enough to mobilise a team to action. Additionally, individual professionals are still trained and motivated to set expectations of performance for themselves and assume responsibility for their own performances. If team performance feedback sessions help develop a shared sense of responsibility and agenda for quality improvement they may serve as an activity more for team building that may enable quality improvement efforts later on.

A key finding was that many non-physician providers found the indicators presented to not be reflective of their role despite our efforts to include indicators expected to involve many different disciplines on a team. Understanding and linking performance feedback to the priorities of the targeted audience and ensuring their buy-in with selected performance indicators may increase the effect of performance feedback on quality improvement [2,6,19,21]. This suggests that more work needs to be done to develop indicators (particularly specific to mental health) that nurses and other health professionals in primary care would embrace as reflective of their work and contribution to the team. In order for feedback to facilitate change, there may also be a need to understand the team context and performance indicators specific to the roles as they are defined and carried out in each team.

### Limitations

The timing of the study, with interviews often following several weeks after presentation of performance data, limits the ability to understand the effect of specific indicators on actual performance improvement. Most respondents were only able to recall general themes of performance feedback rather than specific indicators limiting their comments to domains of performance rather than specific targets or indicators. The small sample size of seven teams potentially limits the transferability of these results. However the teams were fairly diverse. Additionally, many of the themes emerging from the interviews confirmed previous research on physicians alone. Many of the themes recurred across FHT teams and disciplines. However, small numbers of professionals from each discipline limit the ability to achieve saturation of themes within each discipline or highlight differences between disciplines.

## Conclusions

There may be a role for building the capacity for quality improvement of interdisciplinary primary care teams through team feedback sessions on performance. However, as each team may have its own existing functional units best able to receive and act on different elements of performance data, perhaps offering a menu of indicators for teams to select from might allow them to match these to their existing priorities for change and perceived roles. More efforts to develop flexible and interactive performance-reporting structures (that better reflect contributions from all team members) in which teams could specify the information and audience may assist in promoting quality improvement with improved information systems.

## Competing interests

The authors declare that they have no competing interests.

## Authors' contributions

SJ, WH, and MG designed the study. PT and LR collected the data. SJ, MG, PT, LR, CS, GR, and WH analyzed the data. SJ and PT prepared the manuscript. SJ, MG, PT, LR, CS, GR, and WH critically reviewed and accepted the manuscript.

## Pre-publication history

The pre-publication history for this paper can be accessed here:

http://www.biomedcentral.com/1471-2296/12/14/prepub
